# Evaluating the relationship between amyloid-β and α-synuclein phosphorylated at Ser129 in dementia with Lewy bodies and Parkinson’s disease

**DOI:** 10.1186/s13195-014-0077-y

**Published:** 2014-12-01

**Authors:** Marta Swirski, J Scott Miners, Rohan de Silva, Tammaryn Lashley, Helen Ling, Janice Holton, Tamas Revesz, Seth Love

**Affiliations:** Dementia Research Group, Institute of Clinical Neurosciences, School of Clinical Sciences, University of Bristol, Bristol, UK; Department of Molecular Neuroscience, Reta Lila Weston Institute of Neurological Studies and Queen Square Brain Bank for Neurological Disorders, Institute of Neurology, University College London, London, UK

## Abstract

**Introduction:**

Lewy body and Alzheimer-type pathologies often co-exist. Several studies suggest a synergistic relationship between amyloid-β (Aβ) and α-synuclein (α-syn) accumulation. We have explored the relationship between Aβ accumulation and the phosphorylation of α-syn at serine-129 (pSer129 α-syn), in post-mortem human brain tissue and in SH-SY5Y neuroblastoma cells transfected to overexpress human α-syn.

**Methods:**

We measured levels of Aβ40, Aβ42, α-syn and pSer129 α-syn by sandwich enzyme-linked immunosorbent assay, in soluble and insoluble fractions of midfrontal, cingulate and parahippocampal cortex and thalamus, from cases of Parkinson’s disease (PD) with (PDD; n = 12) and without dementia (PDND; n = 23), dementia with Lewy bodies (DLB; n = 10) and age-matched controls (n = 17). We also examined the relationship of these measurements to cognitive decline, as measured by time-to-dementia and the mini-mental state examination (MMSE) score in the PD patients, and to Braak tangle stage.

**Results:**

In most brain regions, the concentration of insoluble pSer129 α-syn correlated positively, and soluble pSer129 α-syn negatively, with the levels of soluble and insoluble Aβ. Insoluble pSer129 α-syn also correlated positively with Braak stage. In most regions, the levels of insoluble and soluble Aβ and the proportion of insoluble α-syn that was phosphorylated at Ser129 were significantly higher in the PD and DLB groups than the controls, and higher in the PDD and DLB groups than the PDND brains. In PD, the MMSE score correlated negatively with the level of insoluble pSer129 α-syn. Exposure of SH-SY5Y cells to aggregated Aβ42 significantly increased the proportion of α-syn that was phosphorylated at Ser129 (aggregated Aβ40 exposure had a smaller, non-significant effect).

**Conclusions:**

Together, these data show that the concentration of pSer129 α-syn in brain tissue homogenates is directly related to the level of Aβ and Braak tangle stage, and predicts cognitive status in Lewy body diseases.

**Electronic supplementary material:**

The online version of this article (doi:10.1186/s13195-014-0077-y) contains supplementary material, which is available to authorized users.

## Introduction

Alzheimer’s disease (AD), Parkinson’s disease (PD) and dementia with Lewy bodies (DLB) are the most common age-related neurodegenerative diseases and together account for 80% to 90% of patients with dementia [1,2]. The pathological hallmarks of AD are extracellular accumulations of amyloid-β (Aβ) as plaques and intracellular aggregates of hyperphosphorylated tau that form neurofibrillary tangles and neuropil threads. The pathological hallmarks of PD and DLB are Lewy bodies and Lewy neurites, composed of α-synuclein (α-syn) [3-5]. Although these defining abnormalities are characteristic and distinct, many dementia cases have mixed pathology: a large proportion of AD patients (>50%) has additional Lewy body pathology in addition to plaques and tangles [6-16]. In Parkinson’s disease with dementia (PDD) and DLB approximately 40% of cases have significant numbers of Aβ plaques and neurofibrillary tangles [17]. Patients with mixed pathology tend to pursue a more aggressive disease course [18], with more pronounced cognitive dysfunction than in patients with pure AD [19-24].

In PD and DLB, the number of cortical α-syn aggregates is significantly higher in patients who have Aβ plaques in the cortex [25,26] and α-syn accumulates within some plaque-associated dystrophic neurites [27]. Transgenic mice expressing both Aβ and α-syn had more Lewy body pathology and more severe deficits in learning and memory than did mice expressing α-syn alone [28]. These studies suggest a synergistic relationship between Aβ and α-syn. However, the reasons for the frequent pathological overlap between AD and Lewy body diseases are poorly understood. A recent meta-analysis of genome-wide association studies of AD and PD did not detect any gene loci that increased the risk of both diseases and concluded that the pathological overlap is likely to result from processes downstream of the susceptibility genes for the individual diseases [29]. α-syn can induce the hyperphosphorylation of tau through the activation of protein kinase A [30] and glycogen synthase kinase 3β [31,32] and, thereby, promote the formation of neurofibrillary tangles. However, it is noteworthy that the most frequent form of pathological overlap between Lewy body diseases and AD is the presence of increased numbers of Aβ plaques in PDD and DLB [25,33], with limited formation of tangles and the interactions between α-syn and Aβ were, therefore, the primary focus of this study.

The predominant modification of α-syn in Lewy body diseases is phosphorylation at Ser129 [34,35]. Approximately 90% of α-syn within Lewy bodies and neurites is phosphorylated at Ser129, compared to 4% in the normal brain [35]. The precise role of α-syn phosphorylation at Ser129 remains unclear: most [36-39], but not all, studies [40-42] suggest that phosphorylation mediates the aggregation and neurotoxicity of α-syn. Irrespective of whether these changes precede the development of Lewy bodies or occur at a later stage, it is well established that pSer129 α-syn levels correlate with disease severity [43-45]. Obi *et al*. [44] found, that in DLB cases with AD pathology, pSer129 α-syn levels correlated strongly with parenchymal Aβ load (as assessed by immunohistochemistry). The aim of our study was to explore this relationship further, in multiple regions of brain from Parkinson’s disease without dementia (PDND), PDD and DLB patients and age-matched controls, by measuring the concentrations of the two major forms of soluble and insoluble Aβ (Aβ40, Aβ42) by sandwich ELISA, as previously [46-49], and of soluble and insoluble α-syn (both total and pSer129 α-syn) also by ELISA. In PD patients we also analyzed the relationship between Aβ, total α-syn, pSer129 α-syn and ante-mortem cognitive function, as indicated by mini-mental state examination (MMSE) scores. Lastly, in SH-SY5Y cells that stably expressed high levels of endogenous α-syn, we assessed the direct influence of different forms of Aβ on the phosphorylation of α-syn at Ser129 *in vitro*.

## Methods

### Case selection

We studied 35 cases of PD (23 PDND and 12 PDD) from the Queen Square Brain Bank (QSBB) for Neurological Disorders, UCL Institute of Neurology, London, and 10 cases of DLB and 17 age-matched controls from the South West Dementia Brain Bank (SWDBB), University of Bristol (Table 1). Protocols for brain banking at the QSBB were approved by the London Multi-Centre Research Ethics Committee (REC reference 08/H0718/54 + 5) and written consent for the use of brain tissue and for access to the medical record for research was obtained from all cases. The South West Dementia Brain Bank had ethical approval from the North Somerset and South Bristol Research Ethics Committee (REC reference 08/H0106/28).

**Table 1:** **Control, Parkinson’s disease non-dementia (PDND), Parkinson’s disease dementia (PDD) and dementia with Lewy bodies (DLB) cases: demographic and clinical data**

	**Control (n = 17)**	**PDND (n = 23)**	**PDD (n = 12)**	**DLB (n = 10)**
Mean age at onset (years) ± SD	N/A	61.1 ± 9.1	58.2 ± 7.7	69.7 ± 7.3
Mean age at death (years) ± SD	79.2 ± 8.7	77.7 ± 6.2	77.85 ± 6.1	77.0 ± 9.0
Mean disease duration (years) ± SD	N/A	16.6 ± 6.7	19.7 ± 6.5	7.3 ± 2.0
Gender (%)	3 (18) female	13 (57) female	6 (50) female	4 (40) female
Mean post-mortem delay (hours) ± SD	37.0 ± 16.6	63.6 ± 27.0	38.2 ± 21.7	28.0 ± 10.9
Mean time to dementia (years) ± SD	N/A	N/A	14.6 ± 6.9	N/A
Median Braak tangle stage (range)	II (0 to III)	II (I to IV)	II (I to IV)	II (0 to III)

N/A, not available; n, number; SD, standard deviation.

All disease cases were diagnosed using widely accepted neuropathological criteria [50,51]. Cases were excluded from the study if they had a neuropathological diagnosis of AD (that is, if histology showed AD neuropathological change that was considered a sufficient explanation for dementia according to the National Institute on Aging-Alzheimer’s Association guidelines for the neuropathological assessment of AD [51] or any other neurodegenerative disease apart from PD or DLB. They were also excluded if neurohistology revealed severe cerebral amyloid angiopathy or other significant cerebrovascular disease.

To assess the possible influence of Aβ-induced phosphorylation of α-syn on cognitive decline in PD patients, our analyses included the time to dementia in patients with PDD, and the score on the MMSE within the last year of life, where available.

In all cases consent had been given for the use of brain tissue and for access to the patients’ clinical records for research.

### Tissue preparation

Brain tissue (200 mg) samples of midfrontal, cingulate and parahippocampal cortex and thalamus were sequentially extracted in 1% NP-40 buffer (140 mM NaCl, 3 mM KCl, 25 mM TRIS, 5 mM ethylenediaminetetraacetic acid (EDTA), 2 mM 1,10 phenanthroline) as previously described for Aβ measurements in human post-mortem tissue [46,48,49,52]. The tissue was homogenized in a Precellys 24 homogenizer (Stretton Scientific, Derbyshire, UK) with 2.3 mm ceramic beads (Biospec, Stratech, Suffolk, UK). The homogenates were spun at 13,000 × g for 15 minutes at 4°C and the supernatant was removed and stored at −80°C. Insoluble material was solubilized by vigorous agitation in 6 M GuHCl, re-homogenized and left for four hours at room temperature (RT) before storage at −80°C.

### Total α-syn sandwich ELISA

Total α-syn level was determined by sandwich ELISA. Mouse monoclonal anti-α-syn antibody (0.5 μg/ml; BD Biosciences, Oxford, UK) was coated onto a NUNC Maxisorp 96-well plate overnight at RT. The plate was washed in PBS/0.01% tween-20 and blocked for 1.5 hours in 1% BSA/PBS. Tissue samples (insoluble and soluble extracts diluted 1:200 in PBS) were added for two hours at RT with constant shaking. The plate was rinsed, tapped dry and biotinylated polyclonal anti-α-synuclein (1 μg/ml; R&D Systems, Oxford, UK) diluted in PBS was added for two hours at RT. The plate was rinsed and tapped dry, streptavidin-horseradish peroxidase (HRP) (1:200, R&D Systems) was added for 20 minutes and, after further washing, chromogenic substrate (TMBS, R&D Systems) was added for 20 minutes in the dark. The reaction was stopped with 2 N sulfuric acid and absorbance at 450 nM read in a FLUOstar Optima plate reader (BMG Labtech, Aylesbury, UK). Total α-syn levels were interpolated from measurements made on serial dilutions of recombinant human α-syn ranging from 62.5 to 0.98 ng/ml (rPeptide, Stratech, Suffolk, UK). Measurements for each sample were repeated in duplicate.

### pSer129 α-syn sandwich ELISA

Mouse monoclonal anti-α-syn antibody (0.5 μg/ml; BD Biosciences) was coated onto a NUNC Maxisorp 96-well plate overnight at RT. The plate was washed in PBS/0.01% tween-20 and blocked for two hours in 1% BSA/PBS. Tissue samples (insoluble extracts diluted 1:99 in PBS, soluble extracts diluted 1:3) were added for five hours at RT with constant shaking. The plate was rinsed and tapped dry and anti-pSer129 α-syn (0.8 μg/ml; Abcam, Cambridge, UK) diluted in PBS was added and left to incubate at 4°C overnight. Following washing of the plate, biotinylated horse anti-rabbit antibody (1.5 μg/ml; Vector labs, Peterborough, UK) diluted in PBS with 0.01% tween-20 was added for one hour at RT. The plate was rinsed and tapped dry, streptavidin-HRP was added for one hour followed by chromogenic substrate for 20 minutes in the dark. The reaction was stopped with 2 N sulfuric acid and absorbance at 450 nM read in a FLUOstar Optima plate reader (BMG Labtech). The concentration of pSer129 α-syn was determined as described previously [53], by interpolation from measurements of serial dilutions (200 to 3.125 ng/ml) of recombinant α-syn that had been phosphorylated at Ser129 by incubating with casein kinase II (see below).

### Specificity of the pSer129 α-syn antibody

We conducted a preliminary study to confirm the specificity of the pSer129 α-syn antibody. Full-length recombinant human α-syn (1 mg/ml; rPeptide, Statech) was incubated with casein kinase I (CKI) (1,000 units, New England Biolabs, Hitchin, UK) or casein kinase II (CKII) (500 units, New England Biolabs, one unit being defined as the amount of CKII required to catalyze the transfer of 1 ρmol of phosphate to 100 μM CKII peptide sequence RRRADSDDDDD in one minute at 30°C) for one hour at 30°C in the presence of 200 μM ATP (New England Biolabs) (protocol adapted from Lee *et al*. [54]; Walker *et al*. [45]). As a control, another sample was treated in the same manner in the absence of either CKI or CKII. Samples were diluted in 1% Tris-buffered saline (TBS) (1:400) and applied to a pre-wetted (in 1% TBS) nitrocellulose membrane and incubated at room temperature for one hour. The membrane was washed in 0.3% Tris-buffered saline with Tween 20 (TBST) then incubated with 10% non-fat milk in 0.3% TBST for one hour at RT with agitation to prevent non-specific binding. After washing the membrane in TBST, primary antibodies (total α-syn, 0.5 μg/ml, BD Biosciences; pSer129 α-syn, 0.8 μg/ml, Abcam; pSer87 α-syn, 200 μg/ml, Santa Cruz, Dallas, TX, USA) diluted in 5% non-fat milk in TBST were applied overnight. The following day the membrane was again washed in TBST and incubated with peroxidase-conjugated secondary antibody diluted in 5% non-fat milk in TBST for one hour at RT with agitation. The membrane was washed and then developed on photographic film using Immobilon™chemiluminescence reagents (Millipore, Danvers, MA, USA) according to the manufacturer’s guidelines.

The pSer129 α-syn antibody labelled α-syn following incubation with CKII, and to a lesser extent CKI, but did not label recombinant α-syn that had not been phosphorylated with CKI or CKII. In contrast, a non-phosphorylation-specific α-syn antibody (BD Biosciences) detected all forms of α-syn, and a pSer87-specific α-syn antibody detected a signal only after incubation of α-syn with CKI (as expected from previous studies by Okochi *et al*. [55] and Paleologou *et al*. [40]). These findings confirmed the specificity of the pSer129 α-syn antibody (see Additional file 1: Figure S1).

### Aβ40 sandwich ELISA

The level of Aβ40 was measured in post-mortem brain tissue samples by sandwich ELISA as described [49,56]. High-binding Costar 96-well plates (R&D Systems) were coated with anti-human Aβ (2 μg/ml; clone 6E10, raised against amino acids 4–7, Covance, Maidenhead, UK) diluted in PBS and incubated overnight at RT. After five washes with PBS containing 0.05% tween-20, the plates were blocked with 300 μL protein-free PBS blocking buffer (Thermo Fisher Scientific, Loughborough, UK) for two hours at RT. After a further five washes, brain homogenate samples (insoluble extracts diluted 1:49, soluble extracts diluted 1:3) and serial dilutions of recombinant human Aβ1-40 (Sigma Aldrich, Dorset, UK) in PBS containing 1% 1,10 phenanthroline (Sigma Aldrich) (to prevent degradation of Aβ [57]) were incubated for two hours at RT with rocking. After a further wash step, the plates were incubated with anti-human Aβ1-40 (1 μg/ml; Covance) for two hours at RT. The antibody was prepared using the Lightning-Link biotinylation kit (Innova Biosciences, Cambridge, UK) according to the manufacturer’s guidelines. After further washes, the plate was rinsed and tapped dry, streptavidin-HRP added for 20 minutes, and chromogenic substrate for 20 minutes in the dark. The reaction was stopped with 2 N sulfuric acid and absorbance at 450 nM read in a FLUOstar Optima plate reader (BMG Labtech). The Aβ1–40 level in the brain tissue samples was interpolated from a standard curve generated by serial dilution of recombinant human Aβ1–40 (Sigma Aldrich) in the range 16,000 to 1.024 nM. Each sample was assayed in duplicate.

### Aβ42 sandwich ELISA

The level of Aβ42 was measured in post-mortem brain tissue samples by sandwich ELISA as outlined above with a few modifications. Anti-human Aβ1-42 (0.5 μg/ml; 12 F4, Covance) was used as the capture antibody. Tissue samples (insoluble extracts diluted 1:9, soluble extracts diluted 1:3) were incubated at RT for four hours. Biotinylated anti-human Aβ (0.1 μg/ml; Thermo Fisher Scientific) diluted in PBS was used for detection and incubated overnight at 4°C. Following washing, rinsing and drying, streptavidin-HRP was added to the plate for one hour and chromogenic substrate for 20 minutes in the dark. Aβ1-42 concentration in brain tissue was interpolated from a standard curve generated by serial dilution (16,000 to 1.024 nM) of recombinant human Aβ1–42 (Sigma Aldrich). Each sample was assayed in duplicate. The Aβ1-42 ELISA did not detect Aβ1-40, and the Aβ1-40 ELISA did not detect Aβ1-42.

### Sandwich ELISA validation

Intra-assay and inter-assay coefficients of variation were calculated for the ELISAs as well as spike and recovery tests (see Additional file 2: Table S3), in which serial dilutions of Aβ40, Aβ42, α-syn or pSer129 α-syn were added to brain homogenates rather than assay diluent. The recovered:added ratio for each added protein (a ratio sometimes termed the response rate) and the correlation between the calculated concentration and measured concentration of added protein were assessed in the insoluble and soluble fractions of the homogenates. The recovered:added ratios of soluble and insoluble pSer129 α-syn (the former well below 1, the latter well above 1) suggests that on addition to brain homogenates, which already contained relatively high baseline amounts of α-syn as well as some pSer129 α-syn, most of the added soluble pSer129 α-syn rapidly aggregated and entered the insoluble fraction in the homogenate. Data from the total α-syn and Aβ42 assays indicated a good recovery rate, with recovery of Aβ40 being 50%. In all of the assays there was a very close linear correlation between the concentration of added protein and the concentration of protein determined by the assay (as shown by the Pearson r and *P* values), enabling valid comparisons to be made between brains and also between cohorts.

### Immunohistochemical assessment of α-syn, pSer129 α-syn, Aβ42 and Aβ40

Formalin-fixed paraffin-embedded sections of mid-frontal, cingulate, parahippocampal cortex and thalamus in all DLB cases were immunolabelled for Aβ1-42 (0.5 μg/ml; Covance), Aβ1-40 (1 μg/ml; Covance), pSer129 α-syn (0.8 μg/ml; Abcam) and α-syn (80 mg/l; Vector Labs, Peterborough, UK) by use of a standard streptavidin-biotin-HRP immunohistochemistry protocol [58]. The extent of immunolabelling of each antigen was measured by field fraction analysis with the help of Image Pro Plus™ software (Media Cybernetics, Marlow, UK) driving a Leica DM microscope with a motorized stage. The software made an unbiased selection of twelve × 20-objective fields and the percentage area immunopositive for the relevant antigen was determined for each section, as outlined previously [59,60].

### Cell culture

SH-SY5Y neuroblastoma cells were transfected with a pCDNA3.1 vector (Life Technologies, UK) containing wild-type human *SNCA* cDNA under the control of a cytomegalovirus (CMV) promoter. Transfection was carried out with TransFast (Promega, Southampton, UK), followed by selection of clones (and their subsequent maintenance) in culture medium containing 0.3 mg/ml G418 (Geneticin, Life Technologies, Paisley, UK). The culture medium for SH-SY5Y cells, either untransfected or stably expressing human wild-type α-syn, consisted of 42% vol/vol Ham’s F12 nutrient mixture (F12) (Sigma) and 42% vol/vol Eagle’s minimum essential medium (Sigma), supplemented with 15% vol/vol fetal calf serum (Sigma), 2 mM L-glutamine (Sigma), 1% vol/vol non-essential amino acids solution (Sigma), 20 units/mL penicillin, 20 mg/mL streptomycin (Sigma) and 250 ng/mL amphotericin B (Life Technologies) at 37°C in 5% CO_2_ (21% O_2_).

### Addition of Aβ to cell cultures

Before treatment with Aβ, the culture medium was replaced with serum-free medium (no fetal bovine serum and no G418) for 24 hous. Aβ solutions were also prepared 24 hours in advance. Stock solutions of 1 mM Aβ42 and Aβ40 (Cambridge Biosciences, Cambridge, UK) in 35% acetonitrile were diluted in serum-free medium at 1 μM and 10 μM. The Aβ was either left overnight to aggregate at 26°C for 24 hours (as previously described [61]) or immediately placed overnight in a −80°C freezer. Aβ (either aggregated or fresh) was added to flasks the following day (10 μM acetonitrile was added to control flasks) and incubated for 24 hours.

### Preparation of cell lysates for sandwich ELISA

Cells were incubated with Dulbecco’s PBS without calcium chloride and magnesium chloride (Sigma-Aldrich) at 37°C for five minutes and then removed from the flask, transferred into a Falcon tube, and spun for three minutes at 13,000 rpm. The cells were washed in PBS and lysed in 100 μl non-denaturing proprietary cell lysis buffer (Sigma-Aldrich, Dorset, UK) according to the manufacturer’s guidelines, and spun at 13,000 rpm for 15 minutes at 4°C. Cell supernatants (soluble fraction) were removed and stored at −80°C until used. A total of 6 M GuHCl (100 μl) was added to the remaining insoluble pellet and left at RT for 1.5 hours (insoluble fraction) before the tube was stored at −80°C.

### Statistical analysis

Whenever possible, parametric statistical tests were used for comparisons between groups (in some cases this required logarithmic transformation of the data to obtain a normal distribution): analysis of variance (ANOVA) with Dunnett’s test for pairwise intergroup comparisons, or repeated measures ANOVA for the analysis of *in vitro* measurements on cells exposed to different concentrations of Aβ during the same experiment. For variables that were not normally distributed even after transformation, the Kruskall-Wallis test was used, with Dunn’s test for pairwise intergroup comparisons. Pearson or Spearman analysis was used as appropriate to assess the correlation between pairs of variables. Statistical tests were performed using GraphPad Prism v5. *P*-values <0.05 were considered statistically significant.

## Results

### pSer129 α-syn correlates with insoluble and soluble Aβ

**Figure 1:**
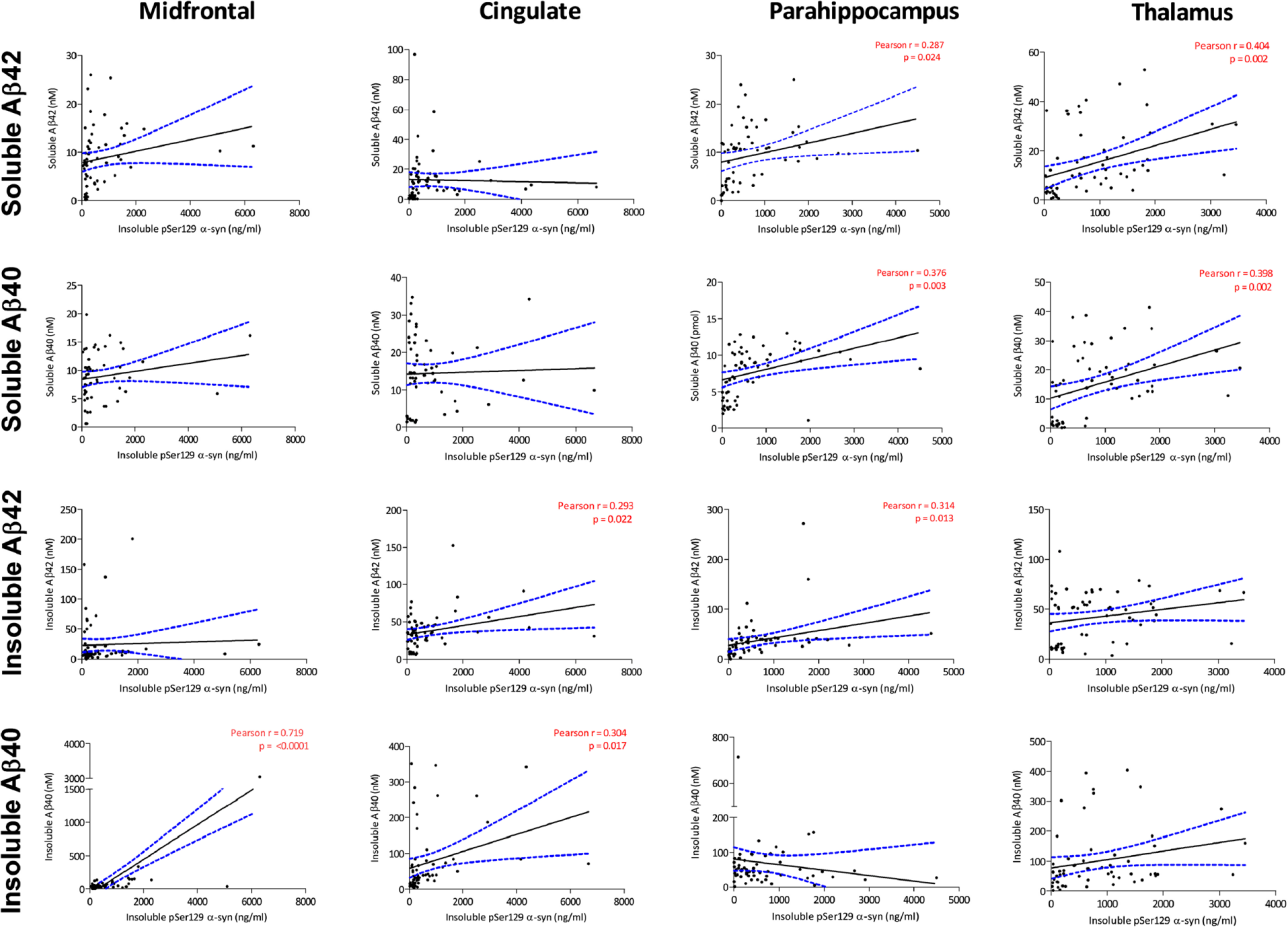
**Correlation between Aβ and insoluble pSer129 α-syn.** Each point represents a separate case. The best-fit linear regression (solid lines) and 95% confidence intervals (interrupted lines) are superimposed. Only significant *P*-values (and associated correlation coefficients) are shown in the figure. Significant positive correlations between insoluble pSer129 α-syn and soluble Aβ42/Aβ40 were found in the parahippocampal (PH) cortex and thalamus (TH). Significant positive correlations were also found between insoluble pSer129 α-syn and insoluble Aβ42 in the cingulate (CG) and PH cortex. In addition, significant correlations were found between insoluble pSer129 α-syn and insoluble Aβ40 in the midfrontal and CG cortex. Aβ, amyloid-β; pSer129 α-syn, alpha-synuclein phosphorylated at serine 129.

**Figure 2:**
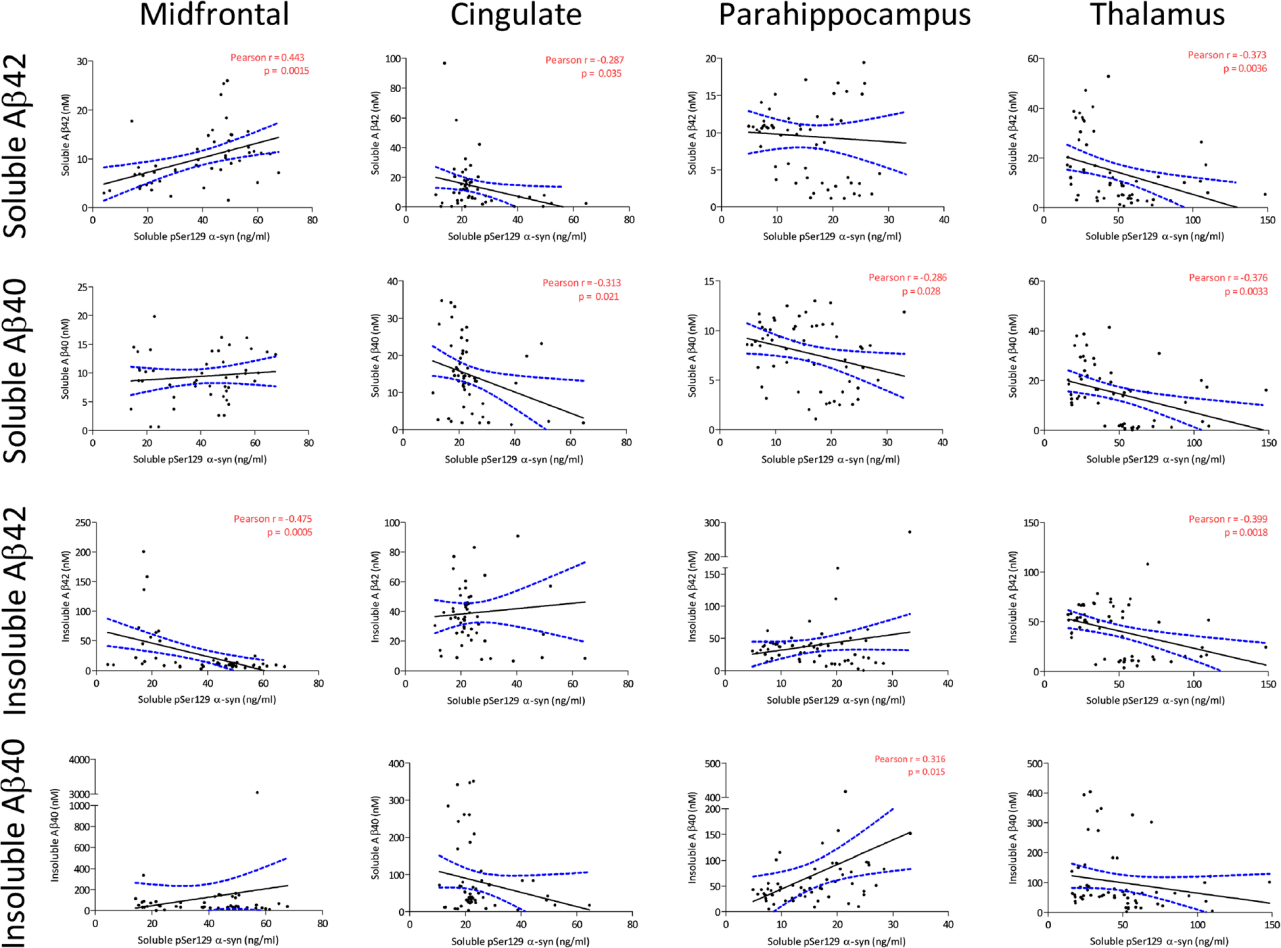
**Correlation between Aβ and soluble pSer129 α-syn.** Each point represents a separate case. The best-fit linear regression (solid lines) and 95% confidence intervals (interrupted lines) are superimposed. Only significant *P*-values (and associated correlation coefficients) are shown in the figure. Significant negative correlations between soluble Aβ42 and soluble pSer129 α-syn were found in the cingulate (CG) cortex and thalamus (TH). In contrast, a significant positive correlation was found between soluble Aβ42 and soluble pSer129 α-syn in the midfrontal (MF) cortex. Significant negative correlations were found between soluble Aβ40 and soluble pSer129 α-syn in the CG, parahippocampal (PH) cortex and TH. Significant negative correlations were also found between soluble pSer129 α-syn and insoluble Aβ42 in the MF cortex and TH. A significant positive correlation was observed between soluble pSer129 α-syn and insoluble Aβ40 in the PH cortex. Aβ, amyloid-β; pSer129 α-syn, alpha-synuclein phosphorylated at serine 129.

In most regions the level of insoluble pSer129 α-syn increased as the levels of Aβ40 and Aβ42 increased (Figure 1, Additional file 3: Table S1), with significant positive correlations between insoluble pSer129 α-syn and soluble Aβ40 and Aβ42 in the parahippocampal cortex (soluble Aβ40: r = 0.376, *P* = 0.003; soluble Aβ42: r = 0.287, *P* = 0.024) and thalamus (soluble Aβ40: r = 0.398, *P* = 0.002; soluble Aβ42: r = 0.404, *P* = 0.002). Significant positive correlations were also found between insoluble pSer129 α-syn and insoluble Aβ42 in the cingulate (r = 0.293, *P* = 0.022) and parahippocampal cortex (r = 0.314, *P* = 0.013) as well as between insoluble pSer129 α-syn and insoluble Aβ40 in the midfrontal (r = 0.719, *P* <0.0001) and cingulate cortex (r = 0.304, *P* = 0.017). Significant negative correlations between soluble pSer129 α-syn and soluble Aβ42 were found in the cingulate cortex and thalamus (Figure 2; Additional file 3: Table S1) (cingulate cortex: r = −0.287, *P* = 0.035; thalamus: r = −0.373, *P* = 0.0036). In contrast, a significant positive correlation was observed between soluble Aβ42 and soluble pSer129 α-syn in the midfrontal cortex (r = 0.443, *P* = 0.0015). Significant negative correlations between soluble Aβ40 and soluble pSer129 α-syn were found in three of four regions (cingulate cortex: r = −0.313, *P* = 0.021; parahippocampal cortex: r = −0.286, P = 0.028; and thalamus: r = −0.376, *P* = 0.0033). Significant negative correlations were also found between soluble pSer129 α-syn and insoluble Aβ42 in the midfrontal cortex and thalamus (midfrontal cortex: r = −0.475, *P* = 0.0005; thalamus: r = −0.399, *P* = 0.0018). A significant positive correlation between soluble pSer129 α-syn and insoluble Aβ40 was also found in the parahippocampus (r = 0.316, *P* = 0.015).

Field fraction analysis of the extent of immunohistochemical labeling of these antigens in the DLB cases revealed a significant positive correlation between pSer129 α-syn and Aβ42 in the mid-frontal region only (r = 0.849, *P* = 0.0019) (see Additional file 4: Table S2). There was only weak, non-significant correlation between the level of these antigens in the brain homogenates and the percentage area labeled in paraffin sections from the corresponding regions in the contralateral cerebral hemisphere.

### Insoluble pSer129 α-syn level correlates with Braak tangle stage

**Figure 3:**
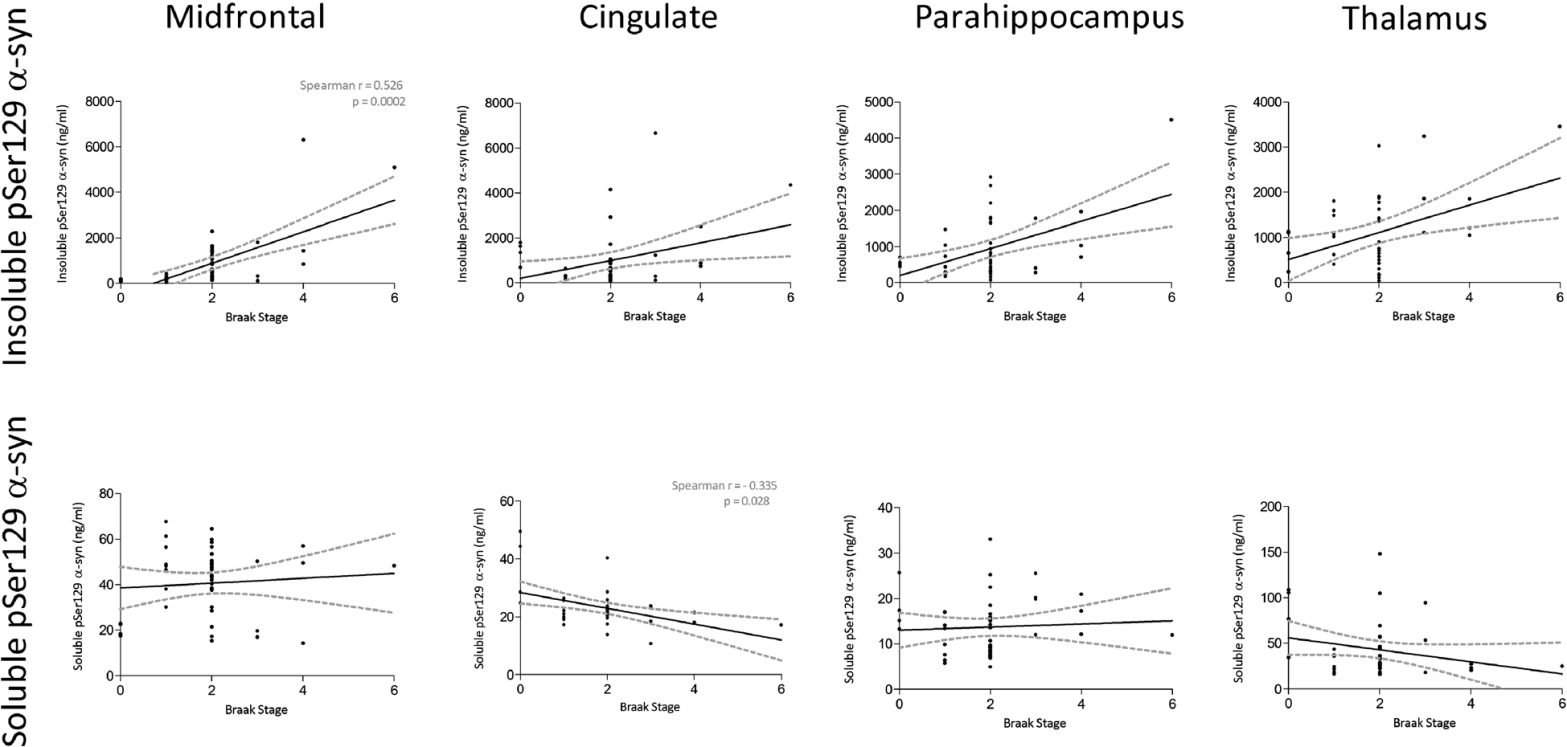
**Correlation between pSer129 α-syn and Braak stage in combined PD (n = 35) and DLB (n = 10) patients.** Each point represents a separate case. The best-fit linear regression (solid lines) and 95% confidence intervals (interrupted lines) are superimposed. Only significant *P*-values (and associated correlation coefficients) are shown in the figure. Insoluble pSer129 α-syn level correlated positively with Braak stage in the midfrontal cortex. Soluble pSer129 α-syn level correlated negatively with Braak stage in the cingulate cortex. DLB, dementia wth Lewy bodes; PD, Parkinson’s disease; pSer129 α-syn, alpha-synuclein phosphorylated at serine 129.

The insoluble pSer129 α-syn level correlated positively with the Braak tangle stage only in the mid-frontal cortex (Figure 3) (r = 0.526, *P* = 0.0002). Soluble pSer129 α-syn levels correlated negatively with the Braak stage in the cingulate region only (r = −0.335, *P* = 0.028).

### Insoluble Aβ is higher in disease groups than controls and in DLB than PD in most regions

**Figure 4:**
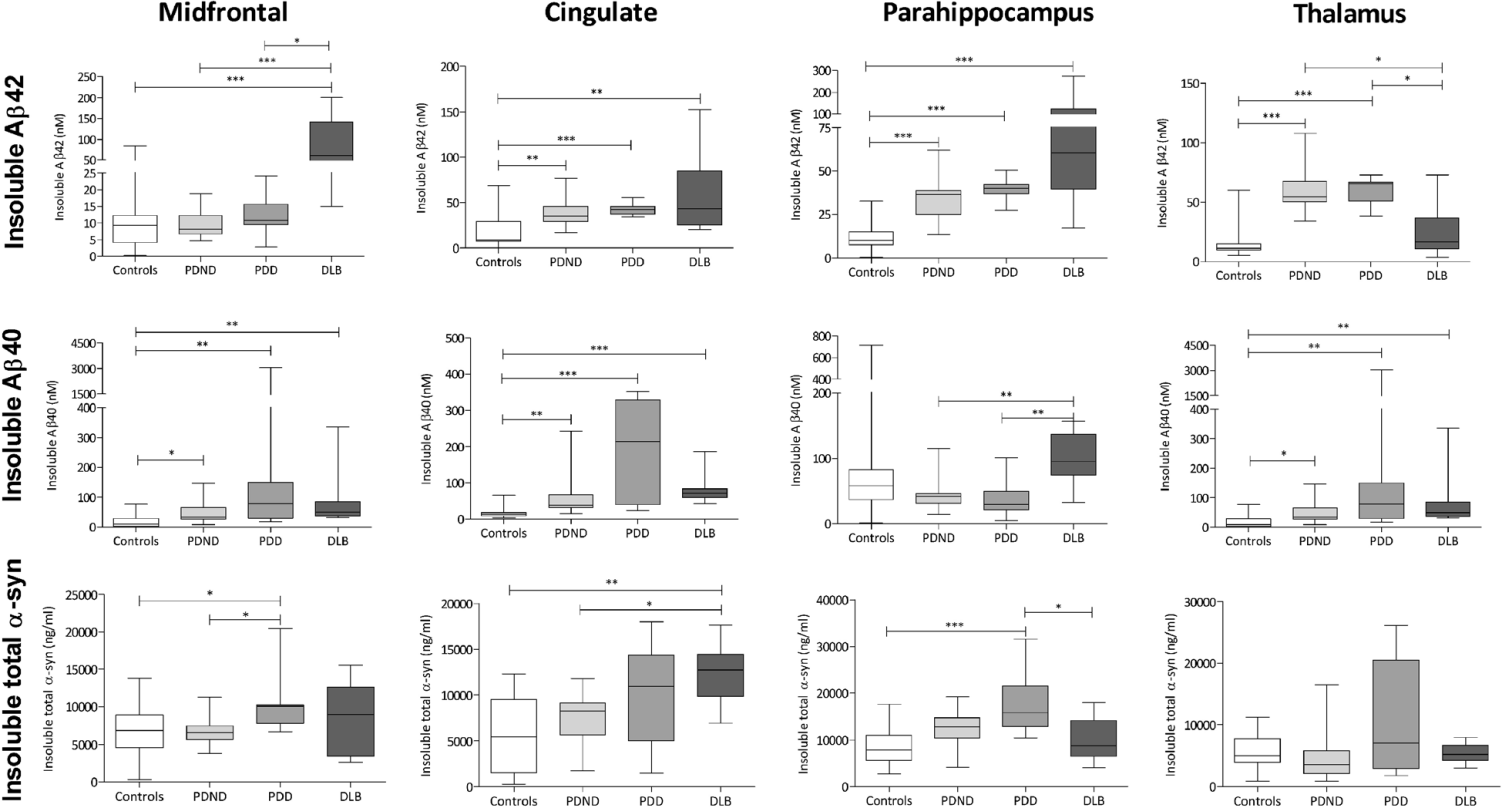
**Insoluble Aβ and α-syn levels in controls, PDND, PDD and DLB.** Box-and-whisker plots indicate the full range, interquartile range and median value in each group. Insoluble Aβ_42_ level was significantly higher in DLB than controls in all regions except the thalamus. It was also significantly higher in DLB than PDND or PDD in the midfrontal region but lower in the thalamus. In all regions except the midfrontal, the insoluble Aβ42 level was significantly higher in both PDND and PDD than controls. The insoluble Aβ40 level was significantly greater in midfrontal and cingulate cortex and thalamus in PDND, PDD and DLB than controls, and in the parahippocampus the level was significantly higher in DLB than in PDND or PDD but not controls. The total insoluble α-syn level was significantly higher in PDD than controls or PDND in midfrontal cortex and higher than in DLB or controls in the parahippocampus. Total insoluble α-syn in the cingulate cortex was significantly higher in DLB than in PDND or controls. No significant differences between groups were observed in the thalamus. Aβ, amyloid-β; DLB, dementia with Lewy bodies; PDD, Parkinson’s disease with dementia; PDND, Parkinson’s disease without dementia; α-syn, α-synuclein.

The level of insoluble Aβ42 in the cingulate and parahippocampal cortex was significantly higher in all disease cohorts than controls (Figure 4). The DLB cohort had a significantly higher level of insoluble Aβ42 in the midfrontal cortex than did any of the other groups. In contrast, the level of insoluble Aβ42 in the thalamus was higher in the PD cohorts than in DLB or controls.

Similar results were observed for insoluble Aβ40, with some regional differences. In the midfrontal and cingulate cortex it was present at a significantly higher level in all disease groups than controls. The level of insoluble Aβ40 in the parahippocampus was significantly higher in DLB than controls.

### Soluble Aβ is higher in disease groups than controls and in dementia groups than PDND in several regions

**Figure 5:**
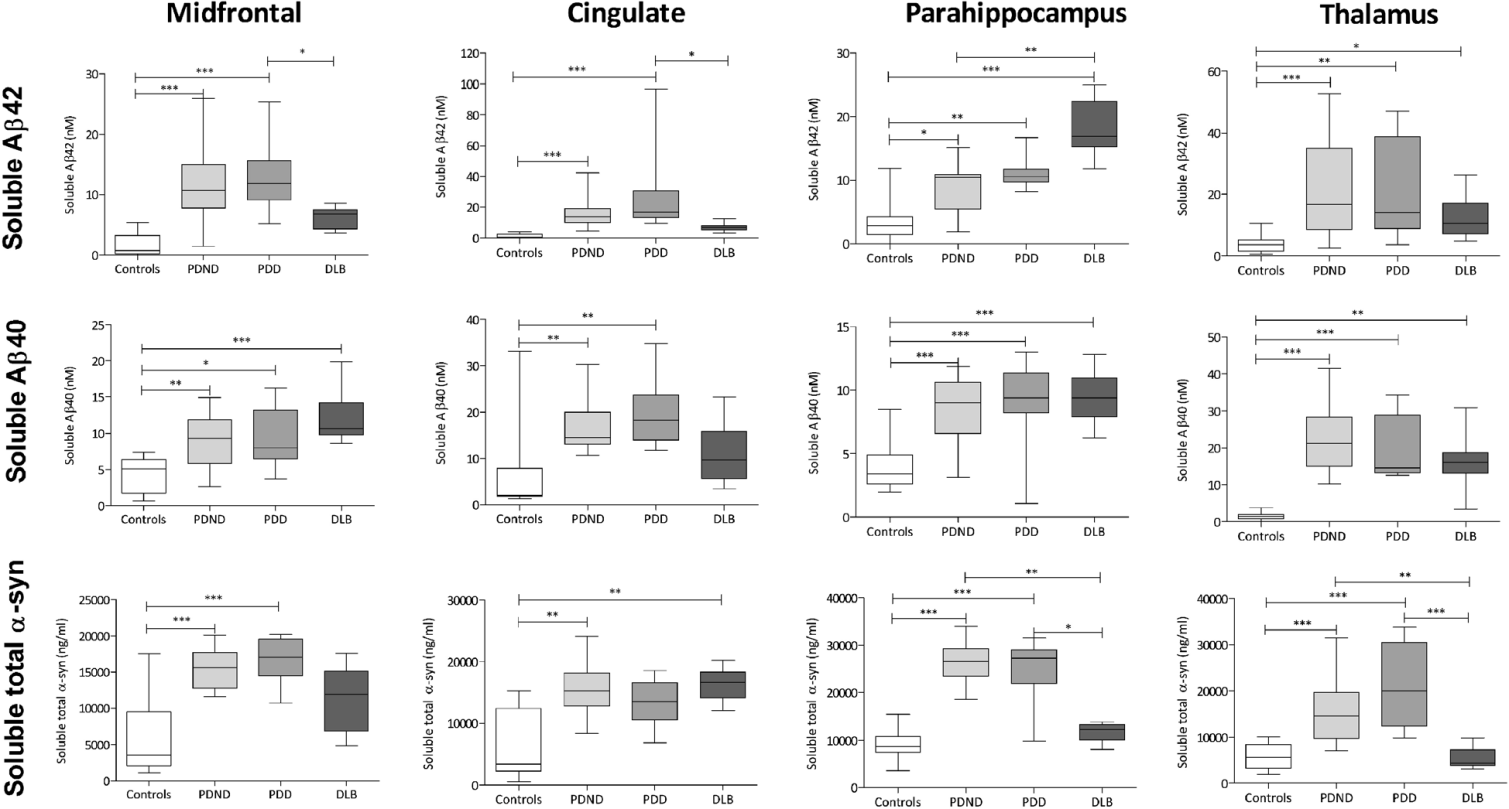
**Soluble Aβ and α-syn levels in controls, PDND, PDD and DLB.** Box-and-whisker plots indicate the full range, interquartile range and median value in each group. Soluble Aβ42 level was significantly higher in PDND and PDD than controls in all regions, and in the midfrontal and cingulate cortex, it was significantly higher in PDD than DLB. In DLB the level of soluble Aβ42 was significantly higher than in controls and PDND in the parahippocampus and higher than controls in the thalamus. The soluble Aβ40 level was significantly elevated in PDND and PDD compared with controls in all regions. The level was also significantly higher in DLB than in controls in all regions except the cingulate cortex. Soluble total α-syn levels were significantly greater in PDND than controls in all regions, and significantly higher in PDD than controls in the midfrontal cortex, parahippocampus and thalamus. *P <0.05, **P <0.01, ***P <0.001. Aβ, amyloid-β; DLB, dementia with Lewy bodies; PDD, Parkinson’s disease with dementia; PDND, Parkinson’s disease without dementia; α-syn, α-synuclein.

The level of soluble Aβ42 was significantly higher in the PD cohorts than controls in all regions (Figure 5). The level in the parahippocampal cortex was significantly higher in DLB than PDND or controls, and in the midfrontal and cingulate cortex it was higher in PDD than DLB. All disease groups had a significantly higher level of soluble Aβ40 than controls in most regions.

### Total insoluble α-syn varies modestly between disease groups

The level of insoluble α-syn in the midfrontal and parahippocampal cortex (Figure 4) was significantly higher in PDD than in PDND or controls whereas in the DLB cohort the level was significantly higher in the cingulate region only. No significant differences were found between groups in the thalamus. No significant differences between controls and PDND were found in any region.

### Total soluble α-syn is higher in PD than controls but lower in DLB than PD

The level of soluble α-syn (Figure 5) was significantly higher in PDND than controls in all regions, and in PDD than controls in the midfrontal and parahippocampal cortex and thalamus. Unexpectedly, the DLB cohort had significantly lower soluble total α-syn than the PD groups in most regions.

### Insoluble pSer129 α-syn is higher and soluble pSer129 α-syn is lower in disease groups than controls

**Figure 6:**
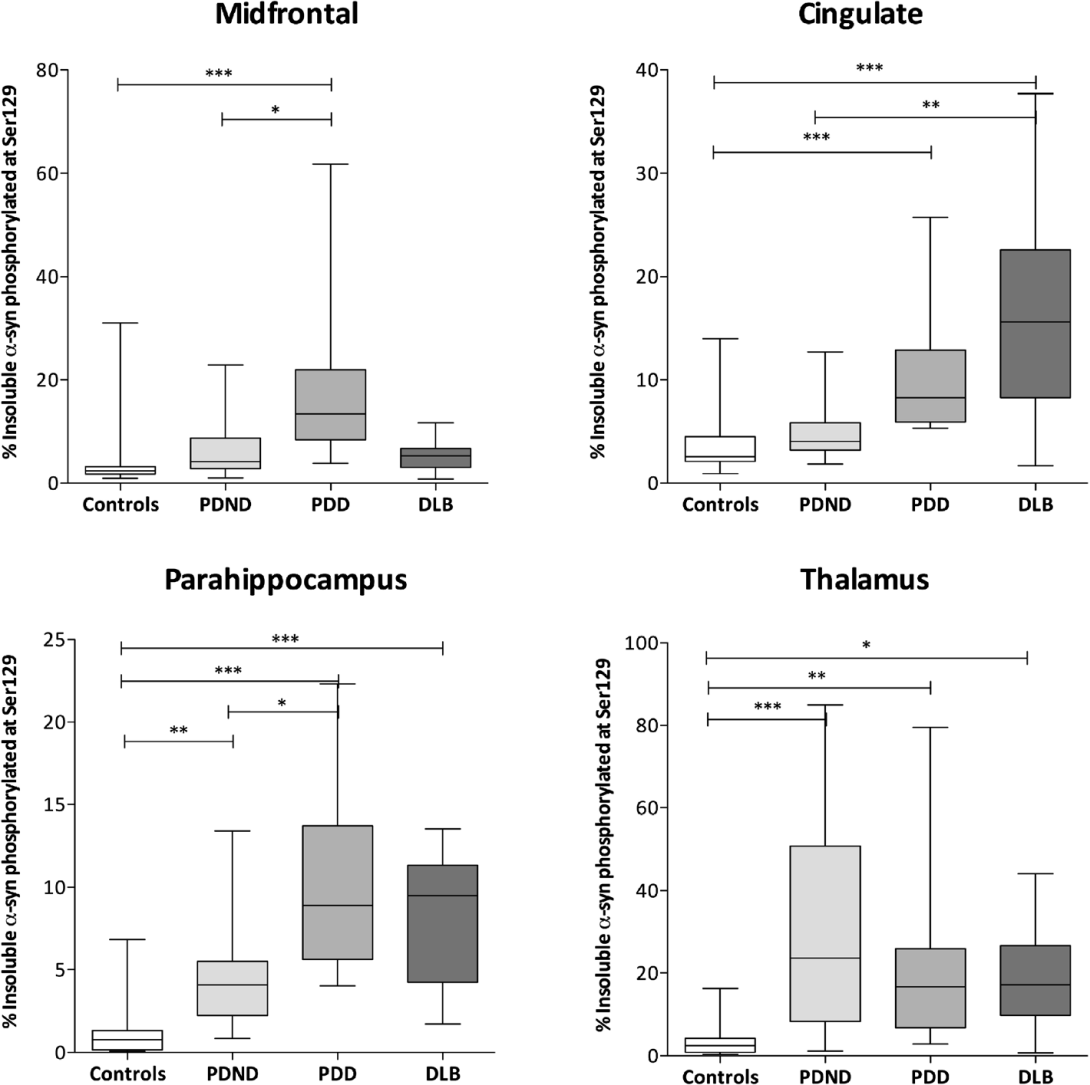
**Percentage of insoluble α-syn phosphorylated at Ser129.** Box-and-whisker plots indicate the full range, interquartile range and median value in each group. The percentage of insoluble α-syn phosphorylated at Ser129 was significantly higher in PDD than controls in all regions and in DLB in regions except the midfrontal cortex. In addition, the percentage was significantly higher in PDD than PDND in the midfrontal and parahippocampal cortex, and in DLB than PDND in the cingulate cortex. In the thalamus and parahippocampal cortex, the percentage of insoluble α-syn phosphorylated at Ser129 was significantly higher in PDND than controls. DLB, dementia with Lewy bodies; PDD, Parkinson’s disease with dementia; PDND, Parkinson’s disease without dementia; α-syn, α-synuclein.

**Figure 7:**
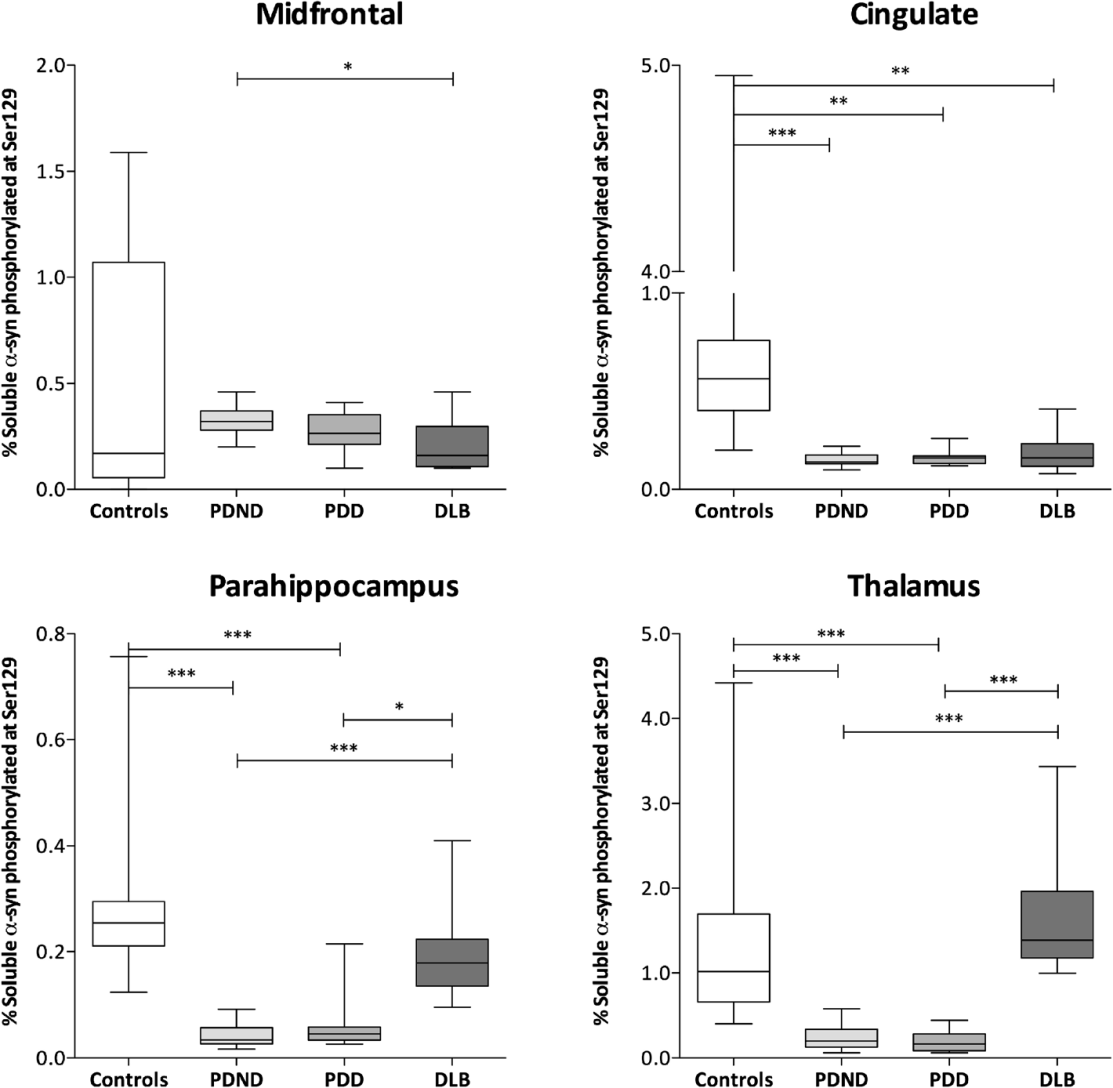
**Percentage of soluble α-syn phosphorylated at Ser129.** The percentage of soluble α-syn phosphorylated at Ser129 was significantly higher in controls than in the PD groups in all regions except midfrontal. The percentage of soluble α-syn phosphorylated at Ser129 was also significantly higher in controls than DLB in the cingulate. Conversely, the percentage was significantly higher in DLB than PDND or PDD in the parahippocampus and thalamus and significantly higher in PDND than DLB in the midfrontal cortex. *P <0.05, **P <0.01, ***P <0.001. DLB, dementia with Lewy bodies; PDD, Parkinson’s disease with dementia; PDND, Parkinson’s disease without dementia; α-syn, α-synuclein.

In most regions, the proportion of insoluble α-syn that was phosphorylated at Ser129 was significantly higher in the PDD and DLB groups than the controls (Figure 6) and in several regions the proportion was significantly higher in both PDD and DLB than PDND. Conversely, in the soluble fraction, the proportion of α-syn that was phosphorylated at Ser129 was significantly higher in controls than disease groups in most regions (Figure 7). Irrespective of cohort, a significantly higher proportion of α-syn was phosphorylated at Ser129 in the insoluble than the soluble fractions (data not shown; *P* <0.001). Absolute protein levels of pSer129 α-syn are displayed in Additional file 5: Figure S2.

### MMSE score correlates negatively with insoluble pSer129 α-syn, total insoluble α-syn and insoluble Aβ42

**Figure 8:**
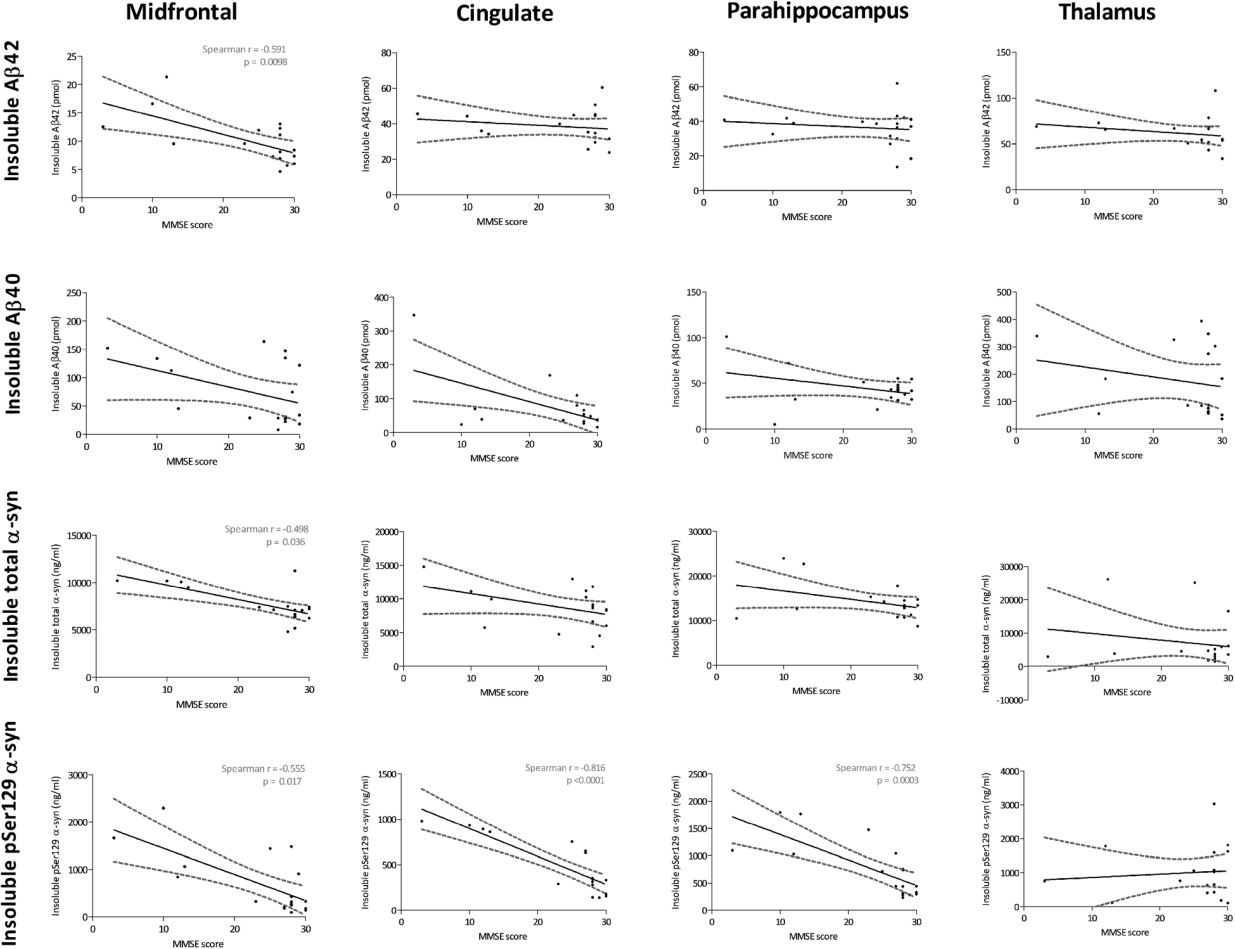
**Correlation between MMSE score and insoluble Aβ or α-syn.** The best-fit linear regression (solid lines) and 95% confidence intervals (interrupted lines) are superimposed. Only significant *P*-values (and associated correlation coefficients) are shown in the figure. In all regions except the thalamus, the level of insoluble pSer129 α-syn showed significant negative correlation with the MMSE score. The score also showed a significant negative correlation with midfrontal insoluble Aβ42 and total insoluble α-syn. Aβ, amyloid-β; MMSE, mini-mental state examination; pSer129 α-syn, alpha-synuclein phosphorylated at serine 129; α-syn, α-synuclein.

In all regions apart from the thalamus, the MMSE score correlated negatively with the level of insoluble pSer129 α-syn (Figure 8) (midfrontal: r = −0.555, *P* = 0.017; cingulate: r = −0.816, *P* <0.0001; parahippocampal cortex: r = −0.752, *P* = 0.0003). Furthermore, in the midfrontal region there was a significant negative correlation between the MMSE score and both insoluble Aβ42 (r = −0.591, P = 0.0098) and total insoluble α-syn (r = −0.498, *P* = 0.036). No significant correlations were observed between MMSE score and soluble Aβ42, Aβ40, total α-syn or pSer129 α-syn. Time to dementia correlated negatively with soluble Aβ40 in this region (r = −0.58, *P* = 0.048) but did not show any other significant correlations (data not shown).

### Aβ treatment induced phosphorylation at Ser129 in α-syn-overexpressing SHSY-5Y cells

**Figure 9:**
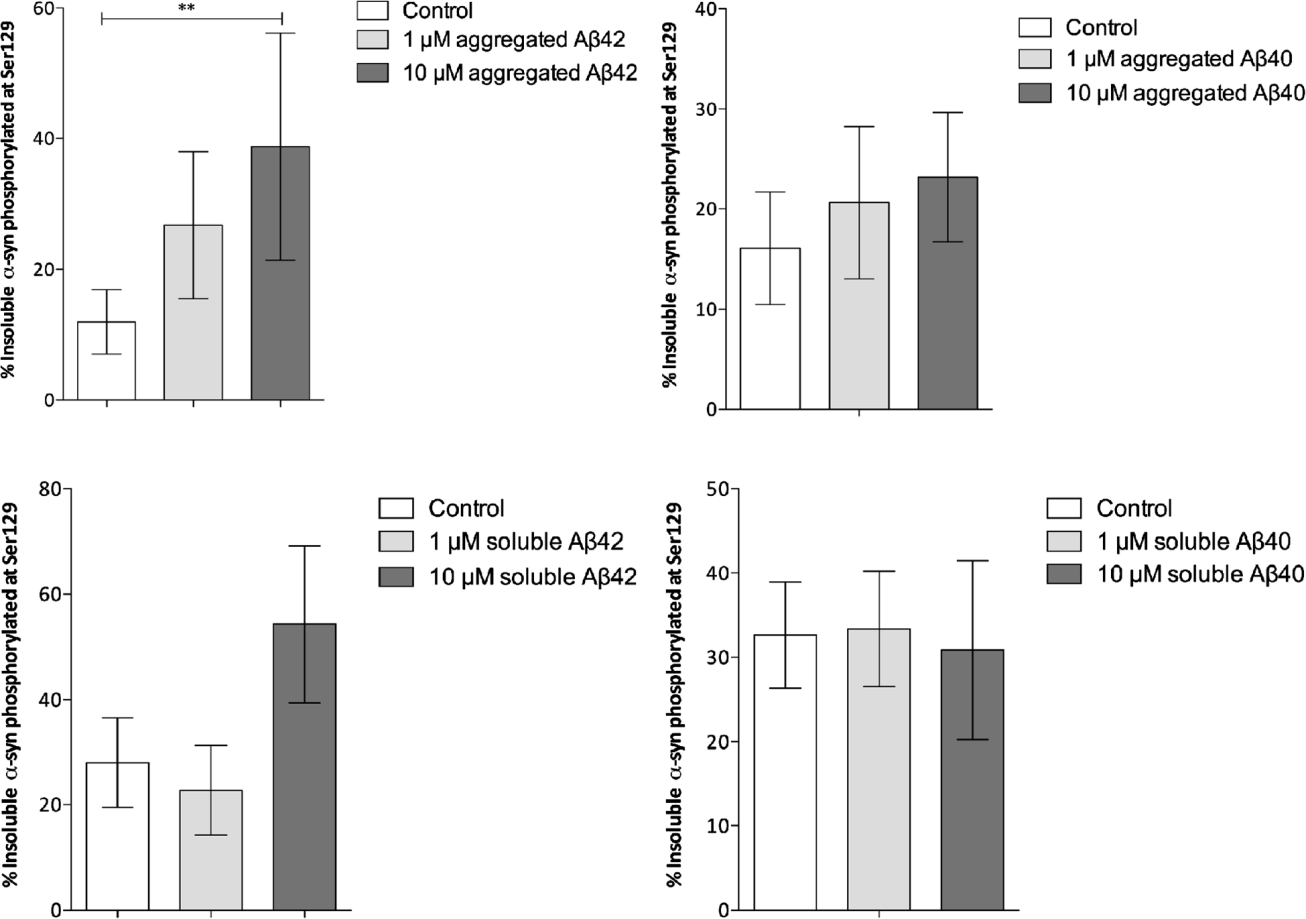
**Percentage of insoluble α-syn phosphorylated at Ser129 after exposure of SH-SY5Y cells to aggregated or soluble Aβ1-42 and Aβ1-40.** The percentage of insoluble α-syn that was phosphorylated at Ser129 was significantly increased after 24 hours exposure of the cells to 10 μM aggregated Aβ1-42 (*P* = 0.009). Ser129 phosphorylation was also higher after exposure to soluble 10 μM Aβ1-42 and aggregated 10 μM Aβ1-40 but the increases did not reach significance. Aβ, amyloid-β.

**Figure 10:**
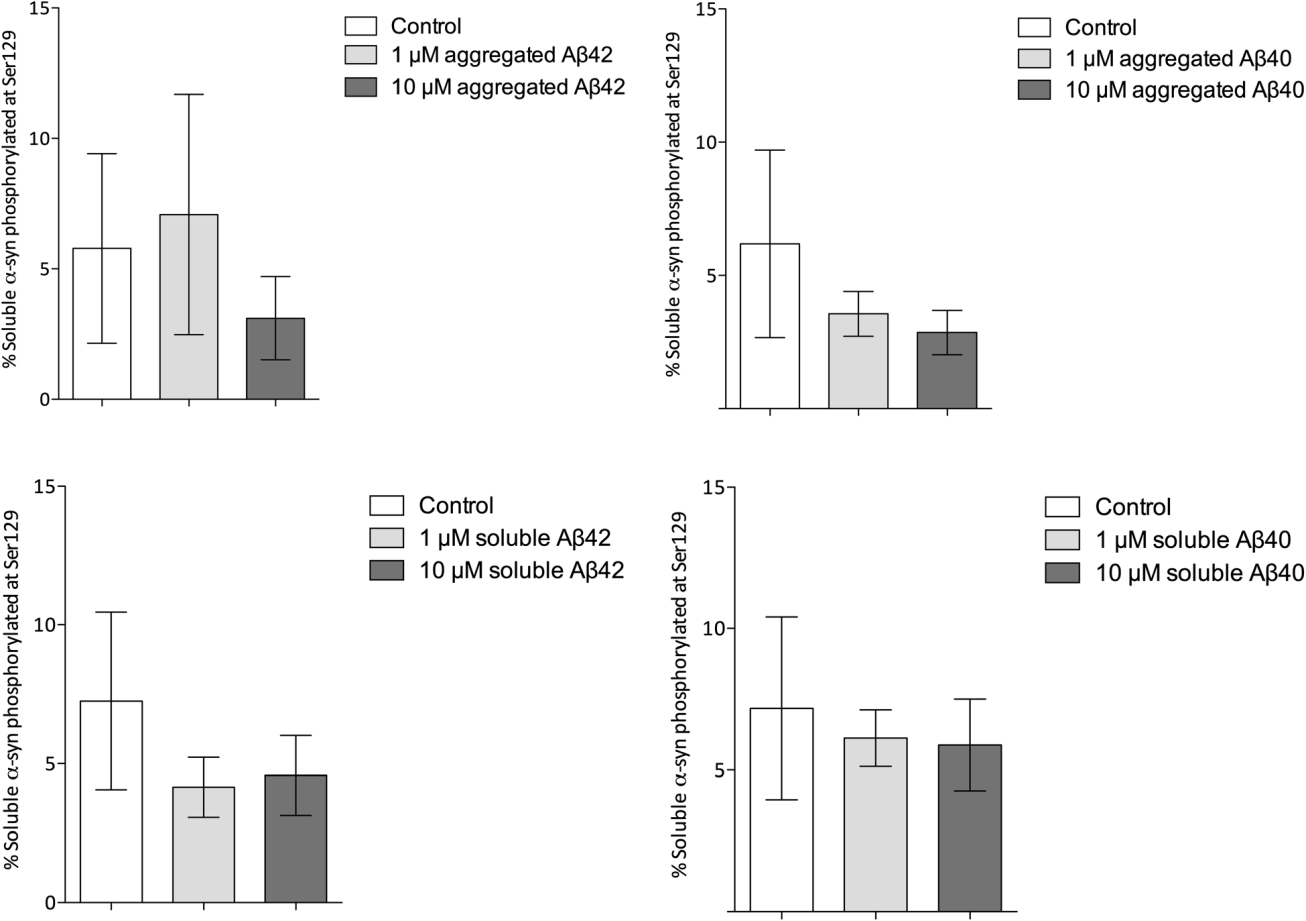
**Percentage of soluble α-syn phosphorylated at Ser129 after exposure of SH-SY5Y cells to aggregated or soluble Aβ1-42 and Aβ1-40.** The percentage of soluble α-syn phosphorylated at Ser129 was much lower than the figure for insoluble α-syn. The percentage tended to fall after exposure to aggregated Aβ1-42 and Aβ1-40, particularly at 10 μM, but not significantly so, and did not change noticeably after exposure to soluble Aβ. The bars show the mean and SE of measurements from five separate experiments. Aβ, amyloid-β.

Exposure of cells to aggregated Aβ1-42 (10 μM) significantly increased the percentage of α-syn in the insoluble fraction that was phosphorylated at Ser129. Ser129 phosphorylation was also higher after exposure to soluble 10 μM Aβ1-42 and aggregated (but not fresh) 10 μM Aβ1-40 but these increases did not reach statistical significance (Figure 9). There was a trend towards a positive correlation (Spearman r = 0.49, *P* = 0.06) between the concentration of aggregated Aβ and the percentage of α-syn phosphorylated at Ser129. In the soluble fraction, the percentage of α-syn that was phosphorylated at Ser129 was much lower and tended to decline after exposure to aggregated Aβ1-42 and Aβ1-40 but not after exposure to fresh Aβ (Figure 10).

## Discussion

Although overlap between AD and DLB pathology occurs much more often than would be expected by chance, the molecular basis is poorly understood. Furthermore, the molecular changes underlying the development of dementia in patients with PD are not fully understood. We found that most parts of the cerebral cortex examined showed: (1) significant correlations between phosphorylation of α-syn at Ser129 and the amount of soluble and insoluble Aβ; (2) significant correlations between phosphorylation of α-syn at Ser129 and Braak stage; (3) higher levels of soluble and insoluble Aβ in PD and DLB than controls, and PDD and DLB than PDND; and (4) a higher proportion of α-syn phosphorylated at Ser129 in PD and DLB than controls, and PDD and DLB than PDND. Our study also showed that the proportion of α-syn phosphorylated at Ser129 correlated with ante-mortem MMSE. Lastly, our *in vitro* studies showed that exposure of SH-SY5Y cells overexpressing wild-type α-syn to Aβ42 significantly increased the proportion of α-syn that was phosphorylated at Ser129. These biochemical studies extend previous findings of a synergistic relationship between Aβ and α-syn and suggest that Aβ, particularly Aβ42, promotes the phosphorylation of α-syn at Ser129.

Our biochemical studies support previous immunohistochemical findings of a positive correlation between insoluble α-syn and Aβ in Lewy body disease [25,26,62-64]. In addition, our finding of a correlation with Braak stage, although more restricted in terms of regions of the cortex, is in keeping with other studies showing associations between α-syn and tangle pathology [7,44] and suggest that there are multiple interactions between Alzheimer-type and Lewy body-type pathology. Deramecourt *et al*. [7] reported that all patients with sporadic DLB had abundant deposits of Aβ42. In addition, in families with autosomal-dominant AD caused by amyloid precursor protein (APP) or presenilin gene mutations, a high proportion of patients show LB pathology at autopsy [65,66]. Furthermore, patients with mixed LB and Aβ plaque pathology have a more aggressive disease course and more pronounced cognitive dysfunction than do patients with pure AD [19,21-23]. Transgenic mice expressing both human Aβ and α-syn also have more severe deficits in learning and memory, and more intraneuronal α-syn inclusions than do mice transgenic for α-syn alone [28]. Other evidence comes from the observation by Kurata *et al*. [67], of enhanced accumulation of both Aβ and phosphorylated α-syn in mice doubly transgenic for mutant APP and presenilin-1 compared to that in mice transgenic for APP alone.

Obi *et al*. [44] demonstrated an association between Aβ and pSer129 α-syn detected immunohistochemically in the human temporal neocortex human tissue, and we found a similar correlation in the mid-frontal cortex. However, it was noteworthy that the correlation between Aβ and pSer129 α-syn was less consistent in different brain regions when we quantified these antigens immunohistochemically than by ELISA, and only a weak, non-significant correlation was demonstrated between the immunohistochemical and biochemical measurements. Several previous studies have highlighted disparities between ELISA and immunohistochemistry [68-70]. Some of these disparities are thought to reflect the effects of formalin fixation and tissue processing on the preservation of antigenic epitopes, and others may relate to a degree of cross-linking of soluble and insoluble proteins, preventing their separate analysis in the fixed, paraffin-embedded tissue. In addition, sandwich ELISAs provide an objective measure of the actual concentration of the analyte in a much larger, more representative volume of tissue than is included in a paraffin section, and relies on a combination of two different antibodies for specificity. Our biochemical methods also allowed us to measure soluble protein. The significant negative correlations between soluble pSer129 α-syn and Aβ are in keeping with an enhanced shift of pSer129 α-syn into the insoluble fraction as a consequence of Aβ. The present findings highlight the importance of combining biochemical assessment with immunohistochemical methods when studying the quantitative relationship between different proteins.

Direct molecular interaction between α-syn and Aβ was demonstrated *in vitro*, by multidimensional nuclear magnetic resonance (NMR) spectroscopy [71]. Aβ42 interacted more strongly than Aβ40 with α-syn, leading to major structural changes to α-syn, and its oligomerization and precipitation within four hours. These findings may be relevant to the observation by Bate *et al*. [72] who observed that Aβ42 (but not Aβ40) enhanced α-syn-induced damage to synapses. Aβ42 more strongly promoted the formation of higher molecular weight α-syn polymers *in vitro* [28]. In keeping with this, we found that Aβ42 level generally correlated more strongly with pSer129 α-syn in human brain tissue extracts than with Aβ40. We also showed that Aβ42 had a more pronounced effect than Aβ40 on the phosphorylation of α-syn in SH-SY5Y cells.

*In vitro* studies have shown that α-syn can be phosphorylated at Ser129 by CKI, CKII [55], several G protein-coupled receptor kinases (GRKs 1, 2, 5, 6) [73], leucine-rich repeat kinase 2 (LRRK2) [74] and Polo-like kinases [75,76]. The levels of CKI and CKII expression are elevated in both AD and DLB [76,77], raising the possibility that these enzymes may be involved in Aβ-induced phosphorylation of α-syn at Ser129, similar to the Aβ-induced phosphorylation of tau [78-80].

More than 90% of α-syn in Lewy bodies and neurites is phosphorylated at Ser129 [34,35]. The importance of pSer129 α-syn was recognized in the Unified Lewy-type Synucleinopathy Staging Scheme of Beach *et al*. [43], based on the abundance and distribution of pSer129 α-syn. We have shown that biochemical measurement of pSer129 α-syn by sandwich ELISA is an excellent marker of Lewy body disease subtype. Previous studies have demonstrated the utility of pSer129 α-syn measurement as a marker of disease stage [43,44] and shown that the level is generally higher in DLB and PDD than in PDND [45]. The accumulation of pSer129 anticipates the development of Lewy body pathology [45,81]. The partitioning and enrichment of pSer129 α-syn in membrane and insoluble brain fractions probably reflects changes in the conformation and solubility of α-syn that promote its association with membrane structures [82-84]. Our data show that exposure of SH-SY5Y cells overexpressing wild-type α-syn to Aβ results in a shift towards insoluble pSer129 α-syn, with a trend towards loss of soluble pSer129 α-syn. In future studies it would be of interest to investigate the distribution of α-syn and pSer129 α-syn following Aβ exposure in this cell model and to determine the enzymes responsible.

We have found a significant negative correlation between the level of insoluble pSer129 α-syn and the MMSE score. This supports previous work suggesting that Ser129 phosphorylation increases the neurotoxicity of α-syn and is detrimental to cognitive function. Sato *et al*. [85] showed that pSer129 α-syn accelerated A53T α-syn-induced neurodegeneration; this effect was abolished by inactivation of G-protein-coupled receptor kinase 6 (GRK6) – responsible for phosphorylation of α-syn at Ser129. In contrast, enhancement of phosphatase activity in α-syn transgenic mice caused a reduction of phosphorylated α-syn, increased dendritic arborization of neurons in the cerebral cortex and reduced astroglial and microglial activation [39]. These morphological effects were associated with improved motor performance. Phosphorylation of α-syn at Ser129 was also shown to reduce α-syn-mediated inhibition of tyrosine hydroxylase, an enzyme involved in catecholamine synthesis; therefore, phosphorylation of α-syn may influence dopamine levels [86]. Although Aβ42, Aβ40 and total α-syn levels in several brain regions all correlated negatively with MMSE score, those correlations were not as strong as that between MMSE score and pSer129 α-syn. Our findings underscore the close association between pSer129 α-syn accumulation and cognitive impairment, and do point to a pathogenetic relationship between Ser129 phosphorylation of α-syn and disease progression.

## Conclusions

Our findings in this study, the first to examine the relationship between α-syn, pSer129, Aβ1-40 and Aβ1-42 levels in human post-mortem brain tissue by sandwich ELISA, support the existence of a pathogenic relationship between the accumulation of Aβ, particularly Aβ42, and the phosphorylation of α-syn at Ser129, increasing the severity of Lewy body disease and the likelihood of dementia. Further investigations are required to determine the precise biochemical pathways responsible for this interaction, the relative contributions of different processes (including Aβ-associated Ser129 phosphorylation of α-syn and α-syn-associated phosphorylation of tau) on the development of combined AD and Lewy body pathology and the progression of neurodegeneration, and also the possible influence of Aβ on other potential sites of α-syn phosphorylation.

## Additional files

Additional file 1: Figure S1. Dot blots demonstrating the specificity of the phosphorylation-specific α-syn antibodies. Blots of recombinant α-syn incubated with distilled water (α-syn) casein kinase I (α-syn + CKI) or casein kinase II (α-syn + CKII) and probed with pan-α-syn (column 1), pSer129 α-syn (column 2) or pSer87 α-syn (column 3) antibody. The pSer129 α-syn antibody labelled α-syn following incubation with CKII, and to a lesser extent CKI, but did not label recombinant α-syn that had not been phosphorylated with CKI or CKII. Labeling with the pSer87-specific α-syn antibody occurs only after incubation of α-syn with CKI. These findings are as predicted from the known patterns of phosphorylation of α-syn with CKI and CKII. Additional file 2: Table S3. Intra-assay coefficient of variation (CV) from insoluble (shaded) and soluble Aβ42, Aβ40, total α-syn and pSer129 α-syn ELISAs. Additional file 3: Table S1. Correlation between levels of Aβ and α-syn in the midfrontal, cingulate and parahippocampal cortex and the thalamus. Additional file 4: Table S2. Correlation between levels of Aβ and α-syn in the midfrontal, cingulate and parahippocampal cortex and the thalamus from immunohistochemistry field fraction analyses. Additional file 5: Figure S2. pSer129 α-syn levels in controls*, PDND, PDD and DLB. Box-and-whisker plots indicate the full range, interquartile range and median value in each group. Insoluble pSer129 α-syn levels were significantly higher in PD groups compared with controls in the midfrontal region. PDD patients also showed significantly higher levels of insoluble pSer129 α-syn compared with PDND and DLB in the same region. All disease groups showed significantly higher levels of insoluble pSer129 α-syn levels compared with controls in the cingulate region. No significant difference in insoluble pSer129 α-syn levels were observed in the parahippocampal cortex and thalamus. Soluble pSer129 α-syn levels were significantly higher in PD groups compared with controls and DLB in the midfrontal region. In contrast, soluble pSer129 levels were significantly higher in controls and DLB compared with PD groups in the parahippocampal region. *Control n numbers varied in the following assays due to limited tissue availability (soluble fraction: total α-syn: midfrontal n = 13, cingulate n = 10, parahippocampal n = 16, thalamus n = 16; pSer129 α-syn: midfrontal n = 5, cingulate n = 12, parahippocampal n = 14; Aβ42: midfrontal n = 13, cingulate n =16; Aβ40: midfrontal n = 9, cingulate n = 14, parahippocampal n = 16).

## Abbreviations

AD: Alzheimer’s disease
ANOVA: analysis of variance
APP: amyloid precursor protein
Aβ: amyloid-beta
BSA: bovine serum albumin
CKI: casein kinase I
CKII: casein kinase II
CMV: cytomegalovirus
DLB: dementia with Lewy bodies
EDTA: ethylenediaminetetraacetic acid
ELISA: enzyme-linked immunosorbent assay
GuHCl: guanidinium chloride
HRP: horseradish peroxidase
MMSE: mini-mental state examination
PBS: phosphate-buffered saline
PD: Parkinson’s disease
PDD: Parkinson’s disease with dementia
PDND: Parkinson’s disease without dementia
pSer129 α-syn: alpha-synuclein phosphorylated at serine 129
QSBB: Queen Square Brain Bank
RT: room temperature
Ser129: serine 129
SWDBB: South West Dementia Brain Bank
TBS: Tris-buffered saline
TBST: Tris-buffered saline with Tween 20
TRIS: tris(hydroxymethyl)aminomethane
α-syn: alpha-synuclein
