# Supplementary figures and images for: Evaluating the relationship between amyloid-β and α-synuclein phosphorylated at Ser129 in dementia with Lewy bodies and Parkinson’s disease
Source: Alzheimers Res Ther. 2014 Dec 1;6(9-9):77. doi: 10.1186/s13195-014-0077-y (PMC4248436; doi:10.1186/s13195-014-0077-y)

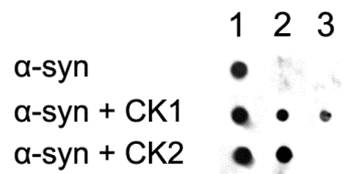

Supplement: Additional file 1: Figure S1. — Dot blots demonstrating the specificity of the phosphorylation-specific α-syn antibodies. Blots of recombinant α-syn incubated with distilled water (α-syn) casein kinase I (α-syn + CKI) or casein kinase II (α-syn + CKII) and probed with pan-α-syn (column 1), pSer129 α-syn (column 2) or pSer87 α-syn (column 3) antibody. The pSer129 α-syn antibody labelled α-syn following incubation with CKII, and to a lesser extent CKI, but did not label recombinant α-syn that had not been phosphorylated with CKI or CKII. Labeling with the pSer87-specific α-syn antibody occurs only after incubation of α-syn with CKI. These findings are as predicted from the known patterns of phosphorylation of α-syn with CKI and CKII. [file 13195_2014_77_MOESM1_ESM.jpeg]

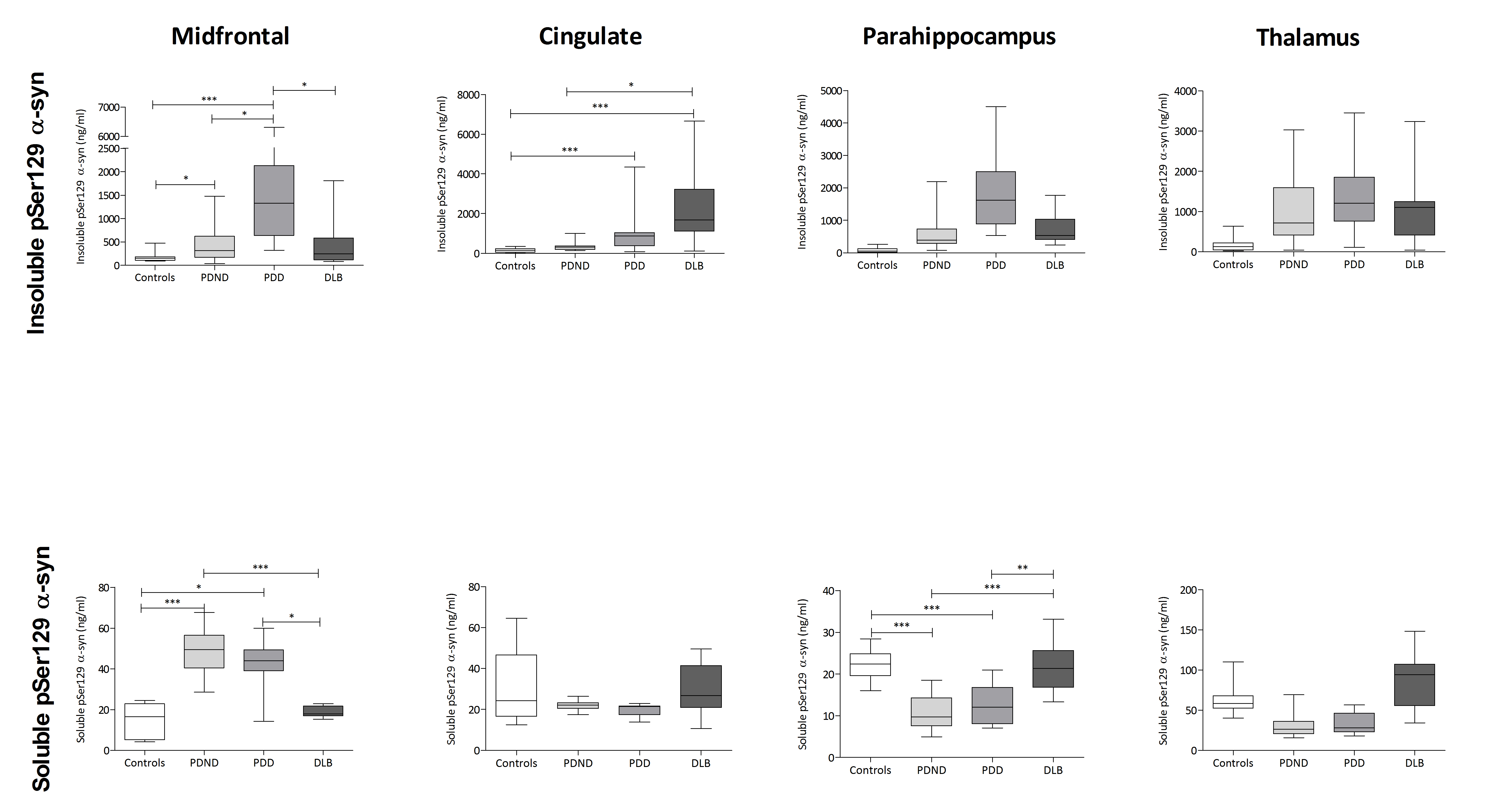

Supplement: Additional file 5: Figure S2. — pSer129 α-syn levels in controls*, PDND, PDD and DLB. Box-and-whisker plots indicate the full range, interquartile range and median value in each group. Insoluble pSer129 α-syn levels were significantly higher in PD groups compared with controls in the midfrontal region. PDD patients also showed significantly higher levels of insoluble pSer129 α-syn compared with PDND and DLB in the same region. All disease groups showed significantly higher levels of insoluble pSer129 α-syn levels compared with controls in the cingulate region. No significant difference in insoluble pSer129 α-syn levels were observed in the parahippocampal cortex and thalamus. Soluble pSer129 α-syn levels were significantly higher in PD groups compared with controls and DLB in the midfrontal region. In contrast, soluble pSer129 levels were significantly higher in controls and DLB compared with PD groups in the parahippocampal region. *Control n numbers varied in the following assays due to limited tissue availability (soluble fraction: total α-syn: midfrontal n = 13, cingulate n = 10, parahippocampal n = 16, thalamus n = 16; pSer129 α-syn: midfrontal n = 5, cingulate n = 12, parahippocampal n = 14; Aβ42: midfrontal n = 13, cingulate n =16; Aβ40: midfrontal n = 9, cingulate n = 14, parahippocampal n = 16). [file 13195_2014_77_MOESM5_ESM.jpeg]
