# Supplementary material for: Evaluating the relationship between amyloid-β and α-synuclein phosphorylated at Ser129 in dementia with Lewy bodies and Parkinson’s disease
Source: Alzheimers Res Ther. 2014 Dec 1;6(9-9):77. doi: 10.1186/s13195-014-0077-y (PMC4248436; doi:10.1186/s13195-014-0077-y)
Supplement: Additional file 2: Table S3. — Intra-assay coefficient of variation (CV) from insoluble (shaded) and soluble Aβ42, Aβ40, total α-syn and pSer129 α-syn ELISAs. [file 13195_2014_77_MOESM2_ESM.docx]

| **Table S3 Intra-assay coefficient of variation (CV) from insoluble (shaded) and soluble Aβ_42_, Aβ_40_, total α-syn and pSer129 α-syn ELISAs** | | | | | | | | | | | |  | |  | |  | | |
| --- | --- | --- | --- | --- | --- | --- | --- | --- | --- | --- | --- | --- | --- | --- | --- | --- | --- | --- |
| CV between mid-frontal samples added in duplicate. | | |  | |  | | |  | |  | |  | |  | |  | | |
|  |  |  |  | |  | | |  | |  | |  | |  | |  | | |
|  | **Aβ_42_ (n=40)** | **Aβ_40_ (n=34)** | **Total α-syn (n=40)** | | **pSer129 α-syn (n=35)** | | | **Aβ_42_ (n=40)** | | **Aβ_40_ (n=25)** | | **Total α-syn (n=40)** | | **pSer129 α-syn (n=35)** | |  | | |
| **CV (%)** | 5.8 | 4.54 | 0.02 | | 4.9 | | | 4.53 | | 4.27 | | 0.04 | | 3.34 | |  | | |
|  |  |  |  | |  | | |  | |  | |  | |  | |  | | |
|  |  |  |  | |  | | |  | |  | |  | |  | |  | | |
| **Inter-assay coefficient of variation (CV) from Aβ42, Aβ40, total α-syn and pSer129 α-syn ELISAs** | | | | | | | |  | |  | |  | |  | |  | | |
| CV based on highest concentration of recombinant protein in five separate plates. | | | | | |  | |  | | |  |  | |  | |  | | |
|  |  |  | |  | |  | |  | | |  |  | |  | |  | | |
| **Assay** | **Aβ_42_** | **Aβ_40_** | | **Total α-syn** | | **pSer129 α-syn** | |  | | |  |  | |  | |  | | |
| **CV (%)** | 17.51 | 14.55 | | 15.19 | | 18.17 | |  | | |  |  | |  | |  | | |
|  |  |  | |  | |  | |  | | |  |  | |  | |  | | |
|  |  |  | |  | |  | |  | | |  |  | |  | |  | | |
| **ELISA spike assays of Aβ42, Aβ40, total α-syn and pSer129 α-syn measured on three human brain homogenate samples** | | | | | | | | | | | |  | |  | |  | | |
| Brain tissue homogenates of cerebral cortex were spiked with serial dilutions of added recombinant protein. | | | | | | | | | | | | | | | |  | | |
| Measurements were made by interpolation against a curve calculated from assay readings on diluent with serial dilutions of added recombinant protein. The baseline concentrations of the relevant endogenous proteins were measured in the homogenates of cerebral cortex. Increases over baseline were attributed to the added recombinant protein, used to determine the amount of protein ‘recovered’. | | | | | | |  | |  | | | |  | |  |  |  |  |
| Values represent the means of measurements on spiked homogenates from three separate brains. Each assay was performed in duplicate. | | | | | | |  | |  | | | |  | |  |  |  |  |
|  |  | | |  | | |  | |  | | | |  | |  |  |  |  |
|  | **Recovered:Added** | | | **Pearson r value** | | | **p value** | |  | | | |  | |  |  |  |  |
| **Aβ42 (soluble fraction)** | 0.76 | | | 0.96 | | | p<0.0001 | |  | | | |  | |  |  |  |  |
| **Aβ40 (soluble fraction)** | 0.5 | | | 0.90 | | | p<0.0001 | |  | | | |  | |  |  |  |  |
| **Total α-syn (soluble fraction)** | 1.03 | | | 0.88 | | | p<0.0001 | |  | | | |  | |  |  |  |  |
| **Total α-syn (insoluble fraction)** | 1 | | | 0.94 | | | p<0.0001 | |  | | | |  | |  |  |  |  |
| **pSer129 α-syn (soluble fraction)** | 0.37 | | | 0.89 | | | p<0.0001 | |  | | | |  | |  |  |  |  |
| **pSer129 α-syn (insoluble fraction)** | 1.41 | | | 0.83 | | | p<0.0001 | |  | | | |  | |  |  |  |  |
