# Supplementary material for: Evaluating the relationship between amyloid-β and α-synuclein phosphorylated at Ser129 in dementia with Lewy bodies and Parkinson’s disease
Source: Alzheimers Res Ther. 2014 Dec 1;6(9-9):77. doi: 10.1186/s13195-014-0077-y (PMC4248436; doi:10.1186/s13195-014-0077-y)
Supplement: Additional file 3: Table S1. — Correlation between levels of Aβ and α-syn in the midfrontal, cingulate and parahippocampal cortex and the thalamus. [file 13195_2014_77_MOESM3_ESM.docx]

| **Table S1** Correlation between levels of Aβ and α-syn in the midfrontal, cingulate and parahippocampal cortex and the thalamus | | | | | | | | |
| --- | --- | --- | --- | --- | --- | --- | --- | --- |
| *Midfrontal* | Insoluble total α-syn | Soluble total α-syn | Soluble pSer129 α-syn |  | *Cingulate* | Insoluble total α-syn | Soluble total α-syn | Soluble pSer129 α-syn |
| Insoluble Aβ_42_ | * |  | *** |  | Insoluble Aβ_42_ |  |  |  |
| Insoluble Aβ_40_ |  |  |  |  | Insoluble Aβ_40_ |  |  |  |
| Soluble Aβ_42_ |  |  |  |  | Soluble Aβ_42_ |  |  | * |
| Soluble Aβ_40_ |  |  | * |  | Soluble Aβ_40_ |  |  | * |
|  |  |  |  |  |  |  |  |  |
| *Parahippocampus* | Insoluble total α-syn | Soluble total α-syn | Soluble pSer129 α-syn |  | *Thalamus* | Insoluble total α-syn | Soluble total α-syn | Soluble pSer129 α-syn |
| Insoluble Aβ_42_ |  |  |  |  | Insoluble Aβ_42_ |  | *** | ** |
| Insoluble Aβ_40_ | * | ** | * |  | Insoluble Aβ_40_ |  |  |  |
| Soluble Aβ_42_ | ** |  |  |  | Soluble Aβ_42_ |  |  | ** |
| Soluble Aβ_40_ |  | ** | * |  | Soluble Aβ_40_ |  |  | ** |
| *p < 0.05, **p < 0.01, ***p < 0.001 (Pearson’s correlation analysis). Shaded fields represent negative correlation, clear fields positive correlation. | | | | | | | | |
